# Lager Beer as Beverage

**Published:** 1889-08

**Authors:** Louis C. D’Homergue


### LAGER BEER AS BEVERAGE.

When lager beer (which signifies a beer which takes time to perfect) was first introduced here, it was hailed as a panacea for the evils of strong drink. When properly made of pure barley, malt and hops and kept for at least a year before tapping, it certainly is one of the best of fermented beverages, and so made and kept, it is comparatively non-intoxicating and gently stimulating. With this idea, our American public, who never do anything by halves, soon acquired a taste for it, and to such an extent that it may now be called a national drink. So great has been and is the demand, that brewers are using materials other than malted barley, such as corn and oats, etc., mixed with barley and hops, by which they accelerate its manufacture, making a sweetish, pleasing, heady beverage, but alcoholic, and the use of this kind of beer, in large daily quantities,with the idea that it is innocuous, has brought on a marked increase of renal complaints. So much so, that many eminent physicians declare such beer more dangerous than the use of whiskey, for the free use of such beer is found to produce a species of degeneration of all the organs ; profound and deceptive fatty deposits, diminished circulation, conditions of congestion and perversion of functional activities, local inflammations of both the liver and kidneys are constantly present. A slight injnry, a severe cold, or a shock to the body or mind, will commonly provoke acute disease ending fatally in a beer drinker. We do not condemn the use of good beer, but it should be used in moderation. Careful people will not drink too much whiskey at a time, because they know it will intoxicate. Well, too much beer at a time acts strongly on the kidneys, and to all those troubled with urinary weakness we say, do not use it.—*From* "*How to Live Long.*" By LOUIS C. D'HOMERGUE, M. D.

